# Health care personnel’s perception of guideline implementation for musculoskeletal imaging: a process evaluation

**DOI:** 10.1186/s12913-020-05272-9

**Published:** 2020-05-11

**Authors:** Ann Mari Gransjøen, Siri Wiig, Kristin Bakke Lysdahl, Bjørn Morten Hofmann

**Affiliations:** 1 Department of Health sciences in Gjøvik, Norwegian University of Science and Technology in Gjøvik (NTNU), Teknologiveien 22, 2815 Gjøvik, Norway; 2 SHARE-Centre for Resilience in Healthcare, Faculty of Health Sciences, University of Stavanger, Kjell Arholmsgate 41, 4036 Stavanger, Norway; 3 Department of Optometry, Radiography and Lighting Design, Faculty of Health Sciences, University of South-Eastern Norway, Box 235, 3603 Kongsberg, PO Norway; 4 Center for medical ethics, University of Oslo, PO Box 1130, Blindern, 0318 Oslo, Norway

**Keywords:** Implementation, Guidelines, Adherence, Practice change, Diagnostic imaging

## Abstract

**Background:**

The increasing complexity and variability in radiology have significantly fueled the need for guidelines. There are many methods for disseminating and implementing guidelines however; and obtaining lasting changes has been difficult. Implementation outcome is usually measured in a decrease in unwarranted examinations, and qualitative data are rarely used. This study’s aim was to evaluate a guideline implementation process and identify factors influencing implementation outcome using qualitative data.

**Methods:**

Seven general practitioners and five radiological personnel from a Norwegian county participated in four focus group interviews in 2019. The data were analyzed using qualitative content analysis, where some categories were predetermined, while most were drawn from the data.

**Results:**

Four main categories were developed from the data material. 1) Successful/unsuccessful parts of the implementation, 2) perceived changes/lack of changes after the implementation, 3) environment-related factors that affected guideline use, and 4) User related factors that affect guideline use.

**Conclusions:**

Our findings show that clinical guideline implementation is difficult, despite the implementation strategy being tailored to the target groups. Several environment- and user-related factors contributed to the lack of changes experienced in practice for both general practitioners and radiological personnel.

## Background

The need for, and use of guidelines have increased significantly with the ever-increasing complexity of medicine in general, and radiology in particular. Moreover, guidelines are responses to unwarranted variability in the services. Many such guidelines target the referrer’s behavior, such as the earlier guidelines in radiology developed by the Royal College of Radiologists, and the American College of Radiologists, among others [1].

Implementation of best care and reducing non-indicated imaging through evidence based guidelines is important since overuse of medical services and inappropriate use of medical services are still problems faced today [2]. These uses of medical services, imaging included, may cause patients more harm than good in the form of incidental findings (which may lead to unwarranted treatment) [3, 4], decreased self-perception of health [5] increased fear-avoidance when imaging results are known by the patients [5, 6], and unnecessary radiation dose [7], with no shown benefits when early imaging is provided [8].

Prevoius guideline implementation strategies for different guidelines aimed towards different parts of the health care system (such as primary care, ambulatory care or emergency rooms) have consisted of publication in relevant journals [9], audit and feedback [10], educational outreach [11], and reminders [12], among others. Such methods have been used to implement the Canadian c-spine rule in radiology [13], to reduce the amount of conventional radiography (CR) performed for low back pain [14] and to reduce the amount of skull radiographs performed [15]. More recently most implementation strategies comprise of educational meetings [16] and providing of educational material [17], support and counseling regarding guideline use [18], audit and feedback [18, 19], guideline-concordant structuring of medical records [17], the use of social media platforms (Twitter, Facebook) [16], and patient education [16].

Lack of lasting impact on outcomes is a common challenge with all of implementation methods to some degree. Guideline adherence tends to be reduced as time goes by [9, 20]. However, the more active approaches (e.g. educational meetings, workshops, audit and feedback) tend to have a greater and more lasting effect than the more passive approaches (e.g, postal dissemination, publication in relevant journals) on dissemination and implementation [21]. The most effective measures however, seem to be multifaceted implementation strategies. One study found that such a strategy led to a reduction in patients referred to secondary care (5% of patients for the multifaceted strategy group vs 10% of patients in the passive implementation strategy group), and cost reduction (approximately 93£ per patient), however patients were less satisfied [17]. A systematic review regarding the reduction of unnecessary imaging and pathology tests found that the three most effective implementations were multifaceted strategies consisting of three components (45% relative reduction), two components (32% relative reduction), and one component (28.6% relative reduction) [22]. Lastly a multifaceted implementation targeted towards prescribing physicians in a Dutch hospital led to a significant reduction in non adherence (30.5% vs 21.8%) [18].

Other implementation strategies that have had an effect on the use of patient treatment or use of diagnostic testing (including imaging) have been targeted implementation including introduction of the guideline and monthly feedback showing an increase in the adherence rate (baseline 28.1%, vs 61.5% for the targeted strategy) [19]. Interventions targeted towards both the clinicians and the families in their care proved more effective than clinicians only (61.9% [34.3%] vs 30%[32%]) [22], modified referral forms reduced imaging by 36.8% [23] and targeted reminders to primary care physicians of appropriate indications for imaging reduces referrals by 22.5% [23].

Implementation outcome is often measured as a reduction in unwarranted examinations or tests, but an implementation process outcome may also be reported, such as self-reported use of guidelines [24]. Qualitative results may supplement and explain quantitative results and can then be used to improve implementation efforts.

A national guideline was developed in 2014 in Norway for diagnostic imaging of non-traumatic musculoskeletal diseases. This guideline contains recommendations regarding which modality (x-ray, computed tomography, magnetic resonance imaging, ultrasound) can be used for the diagnosis of different diseases of the extremities, joints and spine. The Norwegian Directorate of Health’s implementation of this guideline consisted of postal dissemination and publication on their own website, along with publication in an online health library [25]. This had little success in increasing quality and reducing variability of examinations being performed, since most GPs had not used it, and many were not aware of it at all [25]. This led to a need for a second implementation using a different strategy [26]. This implementation consisted of educational meetings and videos, as well as digital access targeted towards General Practitioners (GPs), and shortened versions of the guideline targeted towards GPs and radiological personnel respectively, including the recommendations for the most common complaints in primary care (i.e. pain in the neck, shoulders, lower back and knees). The target groups for this implementation included GPs, radiological personnel and the Norwegian Labour and Welfare Administration (NAV). These groups were chosen since all are involved in making the decision of performing diagnostic imaging. Accordingly, the implementation under scrutiny was strictly a re-implementation, since the guideline had already been implemented.

The aim of this study was to evaluate an implementation process and identify factors influencing implementation outcome using qualitative data analyzed according to the Consolidated Framework for Implementation Research (CFIR) [27] and Implementation Fidelity (IF) [24]. The following research questions guided this study: *Which factors of the implementation strategy were perceived as successful or unsuccessful by GP’s and radiological personnel, and is the use of radiological services perceived as better in accordance with the guidelines? Which factors seem to influence the effect of the implementation?*

Using both CFIR and IF to evaluate the implementation process is unique to this study, which enhances the quality of the evaluation of the implementation. We contribute with insights from the Norwegian setting with the Norwegian musculoskeletal guideline that may be of high relevance for developing implementation strategies in other countries, and for performing evaluations implementation strategies for guidelines in other fields.

## Methods

### Design

This study is the third phase of a project that aims to improve the health service by effective implementation of the Norwegian Musculoskeletal Guideline (NMSG). The first phase explored barriers and facilitators for guideline adherence [25], while the second phase was the development and implementation of the strategy [26]. This phase of the project consisted of a passive part and an active part. In the passive part a short version of the guideline’s recommendations was given to the GPs and radiological personnel, as well as already existing digital resources being provided. These were links to pages where the guidelines were previously published. Lastly, another digital resource were provided to the GPs, which consisted of publishing of the guideline in the Norwegian Electronic Medical Manual (NEL), where it was not previously published [26].

The active part of the implementation strategy consisted of informational meetings with GPs or radiological personnel, where the information was tailored to the attending profession. This meant that GPs were provided with further information regarding conventional x-rays, Computed Tomography (CT), Magnetic Resonance Imaging (MRI) and Ultrasound (US), and which tissues they can depict. For the radiological personnel the tailoring consisted of information regarding how the guideline could be used in their daily work, even though the guideline was primarily aimed towards GPs. During these meetings the participants could ask questions regarding the guideline recommendations. The participants discussed the content of the guideline in the context of their practice, and how they could be relevant for their practice. Educational videos were produced for personnel in the included places of work that could not attend, and other places of work and other places of work where informational meetings were not possible [26]. For more information regarding the informational meetings and videos see the previous study in this project [26].

The current, third phase, was designed as a process evaluation, exploring how the implementation was perceived, and which factors participants experienced influenced the process and outcome.

### Participants and data collection

The study has ethical approval from the Norwegian Social Science Data Services (NSD) (Ref. 48,267, 06 May 2016). The final interview guide and consent form was approved by the NSD in December of 2018. Four focus group interviews to evaluate the implementation strategy were conducted with a total of 12 participants: seven GPs, one board certified radiologist, one trainee in radiology (registrar), and three radiographers during January and February 2019. The groups comprised the following participants: group 1-three GPs; group 2-the radiologist, the registrar and one radiographer (public hospital); group 3-two radiographers (private institution); and group 4-four GPs. Of the twelve participants, nine participants were female and three were male. None of the participants had viewed the videos, but most participants had participated in the meetings. Since all the participants showed some interest for the subject, it could be assumed that these were somewhat more likely to use the guideline. A trained radiographer, with first-hand knowledge of the field and the Norwegian context (AMG), conducted all interviews, AMG is trained in qualitative methods and has experience with qualitative semi structured interviews. But had no prior experience in conducting focus group interviews. Therefore, the research team focused on substantial preparations and discussions before and after the interviews.

Data were collected through semi-structured focus group interviews. This was to prevent leading questions from being asked and to ensure that the topics of interest were covered. The interviews were conducted in three municipalities in one Norwegian county. The participants were chosen on the basis that their place of work was included in the earlier intervention, so they were able to evaluate the implementation strategy. Invitations to the focus groups were extended towards the radiological departments in two different public hospitals, one privately run radiological institution, eight medical centers and NAV, all of which had been included in the previous intervention. Of all the invitees, 13 persons consented to participate. One of those who consented dropped out due to being ill at the time of the focus group was conducted. Some participants had participated in the interviews performed in phase 1, as well as the informational meeting, and some participants had not participated in either. The latter was mainly the case for the radiological personnel. There was good engagement from the participants in all the focus groups, however the engagement may have been somewhat higher in the larger groups, and the discussions between the participants somewhat better.

An interview guide (please see Additional file 1) was developed based on one the CFIR’s five domains and comprised of the following topics from the chosen domain (*reflecting and evaluating*): the intervention, the inner setting, the outer setting, the individual involved and the process used to accomplish the implementation [27].

This study uses the major CFIR domain of *process*, and in particular the construct of *reflecting and evaluating* [27], which refers to acquiring feedback not only about the progress and quality of the implementation, but also about the personal and team debriefing about progress and experience [27]. The questions developed using this framework were used to answer both research questions, i.e., regarding successful and unsuccessful parts of the intervention and factors influencing the perceived outcome of the implementation.

**Fig. 1:**
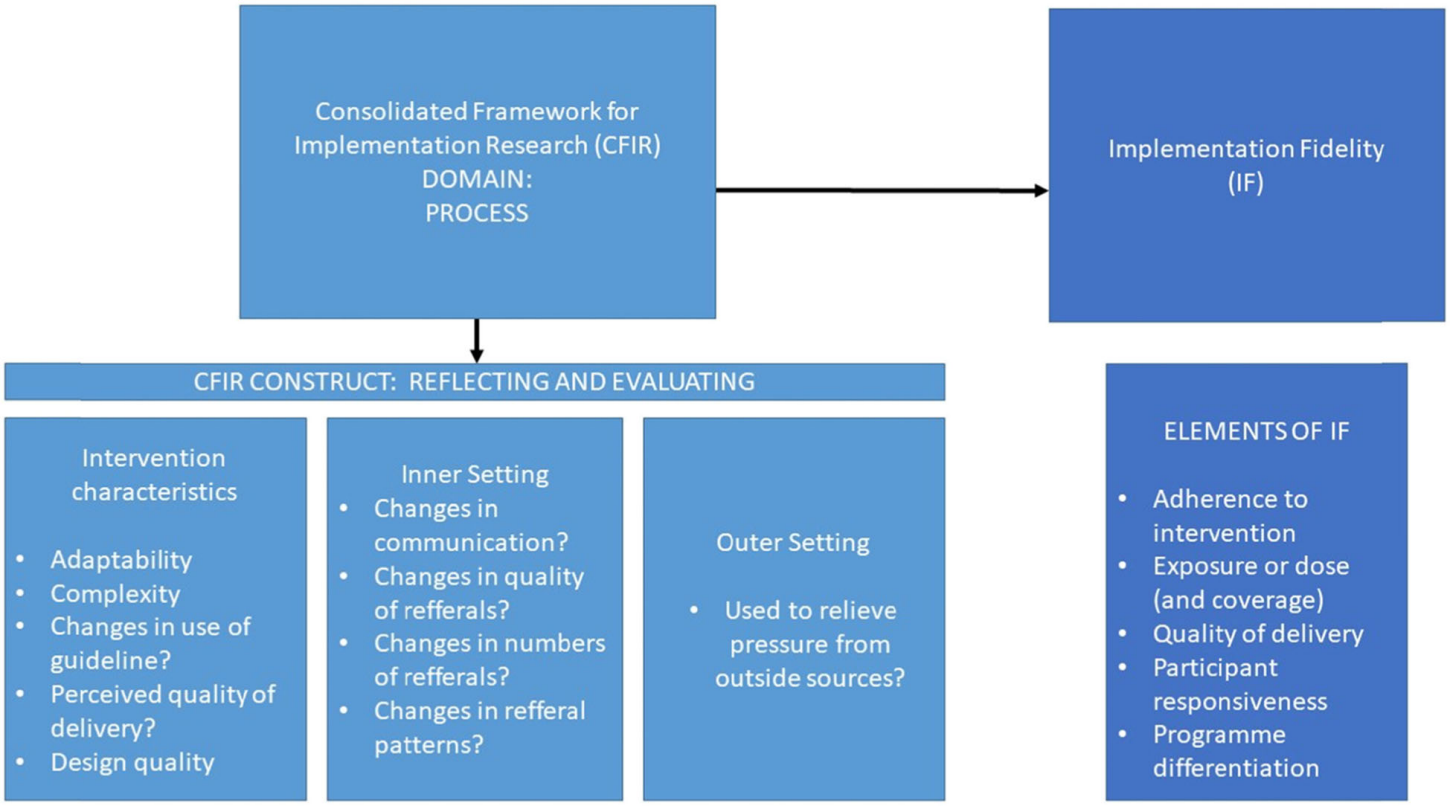
Figure showing an overview of how the construct of reflecting and evaluating was used in this project, and how this is related to implementation fidelity (IF). As can be seen in the figure, some of the points in the intervention characteristics from CFIR overlaps with IF, and can thereby help measure IF. The use of Inner setting and Outer setting from CFIR was used as an indication of adherence, and could thereby help measure IF

Additionally, the interview questions developed using CFIR were also used to measure IF, together with questions based on the IF framework. IF refers to the degree to which the implementation is delivered as intended [24]. Many factors can affect the degree of IF, and IF affects the intervention’s outcome. Potential moderators affecting the adherence are the intervention’s complexity, the facilitation strategies, delivery quality and participant responsiveness [24]. In this case the questions based on IF also helped answer the same research questions as the questions based on CFIR. To the best of our knowledge this is the first study to have explicitly used CFIR and IF in this type of tandem, where the construct of *reflecting and evaluating,* more specifically the points of *intervention characteristics, inner setting* and *outer setting* to help measure IF, and thereby helps improving the evaluation of the implementation. These points from CFIR is suited for this task since it also evaluates the quality of the intervention, and changes in *inner setting* or *outer setting* can be an indicator of adherence. See Fig. 1 for more detail.

The interview guide also covered characteristics of the intervention, and of the perceived quality of the educational materials provided, as well as the intervention’s inner and outer setting. One example of questions used in the interview guide for the former are: *How do you feel the information given and the written material have influenced your knowledge in this particular field?* An example of a question used in the interview guide regarding the outer setting is: *Have you used the guideline in meetings with demanding patients?*

All interviews were conducted at the participants’ workplaces. The interviews lasted approximately 45 min, and ranged from 40 to 60 min, and mean duration was 49 min. See Additional file 1 to see the full interview guide.

Data material can be provided upon reasonable request.

### Analysis strategy

The data material comprise four semi-structured focus group interviews. All interviews were tape recorded and transcribed verbatim. The data were analyzed using Qualitative Content Analysis, which consists of five steps: 1)building a coding frame, 2)segmentation, 3)trial coding, 4)evaluating and modifying the coding frame, and 5)main analysis [28]. In the building of the code frame some of the categories and their respective subcategories were pre-determined. However, most of the categories and subcategories were drawn from the data, meaning the building of the coding frame was partially deductive.

All authors contributed to all steps of the analysis to avoid bias, ensuring a fitting coding frame was built and ensuring that the interpretation reflected the material.

## Results

The coding frame that was built in the first step of analysis, and used in the further analysis of the material, consisted of four categories, each consisting of three to five subcategories. Two of the categories relates to the intervention (parts that were perceived as successful/unsuccessful) and any perceived changes associated with guideline adherence, or lack thereof. The two last main categories relates to factors that affected guideline adherence. All categories and subcategories includes the viewpoints from both the GPs and the radiological personnel. For an overview of the categories and subcategories, see Table 1.

**Table 1:** Overview of the coding frame with main categories and their subcategories

Main Categories	Subcategories	Quotes
**Elements of the implementation perceived as successful/non-successful (3.1)**	Informational meetings	*“So I have to say that I see what you have said [during the informational meeting]as a very positive contribution towards me making better assessments of what are necessary examinations for my patients”*
	Digital access to the guideline	*“I mean it is NEL we mostly use, and many of the guidelines are incorporated there.”*
	Physical copy of the short version of the guideline	*“I had it for a while too, but things like these, they disappear.”*
**Perceived changes, or lack of changes, in practice after the implementation (3.2)**	Changes in use of the guideline	*“I have used it a little, to show patients that there is actually a cost for us to perform diagnostic imaging. Or make an assessment of when to perform diagnostic imaging or not”*
	Changes in referral pattern	*In general the number of x-rays we perform have decreased lately, but if you go to [private institution] I don’t think you will see the same trend there”*
	Changes in justification and quality of referrals	*“[…]where I may still refer to unnecessary MRI examinations of the shoulders and things like that, where the examination wont give much […]”*
	Changes in communication between primary and secondary care	*“But the answers with the results [of the examination]is as before, there is no increase in feedback or anything like that”*
	Changes in communication with Norwegian labor and welfare administration	*“But the problem is more that NAV generally has a high demand towards us to refer to examinations, and our clinical examination is not enough for them.”*
**Environment related factors affecting guideline use and adherence (3.3)**	Organization and placement of responsibility	*“So we don’t have a regulatory body, and the way the system works today there isn’t any possibility to have a regulatory body for x-ray referrals either.”*
	Lack of forum for direct communication	*“No, that’s difficult since we don’t have a system where we can give this kind of feedback without rejecting the referral completely”*
	Patient as a customer	*“You have so much power as a patient today, for a referrer…You can rate them [the referring GPs] by how happy you are, and those reviews are public online…”*
	Private actors in the radiological field	*“Well that is what is being advertised by some private actors. Make sure you are healthy”.*
	Public media attention	*“So the patients will see so much scare mongering in the media, especially [tabloids].*
**User related factors affecting guideline use and adherence (3.4)**	Professional autonomy	*“After all, that’s what clinical judgement is in primary care, to deviate from guidelines and touch and clinically assess the patient”*
	Complexity of the decision-making process	*“It’s not just that knee in a way, you have to consider the entire person with their medical and family history […] and situations like that is what makes you deviate from the guidelines.”*
	Atitudes toward guidelines	*“But it hasn’t been any focus on [the guideline] from the higher ups anyway. We may not be that good at referring to these kind of guidelines in general.”*

### Elements of the implementation perceived as successful or non-successful

Most participating GPs perceived the *informational meetings* that were arranged as positive, albeit with too much information to be able to handle the details. For this reason the *informational meetings* were seen as successful as a reminder of the existence of the guideline and as a repetition of the guideline recommendations, as described by one GP.

*“So I have to say that I see what you have said [during the informational meeting]as a very positive contribution towards me making better assessments of what are necessary examinations for my patients”*

As only few of the radiological personnel in both the hospital and the private institution visited participated in the *informational meetings*, very few of the radiological personnel interviewed could comment on the meeting. However, those who had participated deemed the meetings as positive in regards to increasing awareness of the guideline’s existence. Nonetheless, they thought it more important that the referrers – rather than themselves – had received the information of the guideline, and that his could lead to greater changes in the use of diagnostic imaging.

The GPs were also satisfied with the *digital access to the guideline*, through the Norwegian Electronic Handbook for doctors (https://legehandboka.no/). This is the digital resource most widely used by GPs when searching for information, such as guideline recommendations regarding certain diseases. Radiological personnel do not use the Electronic Handbook for doctors and had not used the links provided with the physical short version, and they found it somewhat difficult to find the guideline through online searches.

The *access to a physical short-version* of the guidelines recommendations was perceived as the least successful part of the implementation. Most of the GPs had used them for a while immediately after receiving them, but these papers had gone missing quite soon after the implementation.

*“I had it for a while too, but things like these, they disappear.”*

None of the participating radiological personnel was aware of the existence of the physical short versions, which the participants explained by the non-optimal placement of these short versions in a room that was not often used. The videos were not used by the participants, and where thereby not mentioned during the focus groups.

### Perceived changes, or lack of changes, in practice after the implementation

All changes that are listed in this section are not objectively measured, as these are the perceived changes of the participants. The implementation led to perceived *changes in the use of the guidelines* to some extent. Many chose not to consult the guideline in their daily work, because they perceived their practice complied with the current guideline recommendations, as presented during the informational meetings. This meant that the guideline was not used as a decision-making tool, but rather as a tool in discussions with patients:

*“I have used it a little, to show patients that there is actually a cost for us to perform diagnostic imaging. Or make an assessment of when to perform diagnostic imaging or not”*

As far as the participating radiological personnel knew, the guideline had not been used in their departments after the implementation. There was, however, some agreement that they experienced a slight decrease in the numbers of examinations performed in their respective departments. The participants could not remember if this decrease happened in conjunction with the implementation.

*“In general the number of x-rays we perform have decreased lately, but if you go to [private institution] I don’t think you will see the same trend there”*

Some of the GPs responded that they had started to wait longer with some patients before referring them to diagnostic imaging, reflecting a *change in referral pattern*. However, some participants also felt that they still referred for unwarranted examinations, in the sense that they most likely would not change a patient’s diagnosis or treatment. The radiological personnel did not perceive any changes in the quality of the referrals received in their respective departments (hospital/private institution).

The analyses show no perceived changes in the *communication between the GPs and radiological departments*, or in the *communication with the Norwegian Labor and Welfare Administration (NAV).*

### Environment-related factors affecting guideline use and adherence

Both GPs and radiological personnel argued that the *organization and placement of responsibility* in the health care services could make it difficult to adhere to guidelines, especially for radiological personnel. The radiological personnel frequently mentioned two points. First, the referrals for conventional x-ray examinations usually were not assessed by a radiologist in most departments, which limits the possibilities for guideline use and adherence.

Second, the drop-in system used by many radiological departments also made guideline adherence difficult. This system allows the patient to go directly to the radiological department with a referral from their GP without a booked appointment and have the examination performed immediately. This statement from one of the radiological personnel shows this lack of a system for x-ray referral assessment:

*“So we don’t have a regulatory body, and the way the system works today there isn’t any possibility to have a regulatory body for x-ray referrals either.”*

Both of these points were seen as problematic because the radiographer is the first to make an assessment of the referral in both instances, when the patient had already arrived in the department. This complicated the assessment and any potential change or declining of the referral.

We found that GPs perceived pressure from other professions (e.g, physiotherapists and chiropractors) and patients as a challenge to guideline adherence, i.e. *patient as a customer* perception. For example, younger GPs could be concerned about bad reviews online and patients changing their GP if they did not receive a referral. Radiological personnel also talked about these challenges and sympathized with the GPs’ perceptions. The statement made by one of the radiological personnel showed that both groups received this as a problem:

*“You have so much power as a patient today, for a referrer…You can rate them [the referring GPs] by how happy you are, and those reviews are public online…”*

Radiological personnel did not talk about the same type of pressure; rather they experienced a pressure to perform the examination requested by the referrer. Moreover, they talked about how they worked at a service department that was supposed to accommodate the other departments in the hospital and referrers from primary care.

Some GPs feared that the risk of *public media attention* (such as newspaper articles) impelled an increased demand for diagnostic imaging in the population. Another problem mentioned by many of the GPs were the *private actors in the radiological field*, who market their services to the population, thereby creating a perceived need for diagnostic imaging among the patients.

Finally, both GPs and radiologists perceived a *lack of a forum for direct communication* about the referrals as an obstacle for assuring guideline adherence and quality of referrals. The statement by one of the radiological personnel demonstrates this:

*“No, that’s difficult since we don’t have a system where we can give this kind of feedback without rejecting the referral completely”*

They were talking about technological systems in that quote. It was also mentioned that the electronic referral system used by many hospitals and privately run institutions could be the ideal place to have such a feature, enabling feedback about referrals more easily that today.

GPs missed feedback from radiological personnel to correct their practice, while radiological personnel missed having an effective means of asking questions about referrals and giving feedback to the referrer.

### User related factors affecting guideline use and adherence

Our study found that most GPs perceived that their *professional autonomy* and clinical judgement had more weight than guideline adherence when assessing a patient for potential diagnostic imaging, illustrated by this GP’s statement:

*“After all, that’s what clinical judgement is in primary care, to deviate from guidelines and touch and clinically assess the patient”*

Radiological personnel also argued that *professional autonomy* was important when assessing referrals and deciding whether a need existed for a modality deviating from a referrers request.

Our findings also show that GPs considered the *decision-making-process to be complex,* during which not only a patient’s current symptoms needed to be taken into account, but also a patient’s previous history, family history, and other relevant factors. These combined factors often made guideline adherence difficult, since the guideline recommendations often do not take these factors into consideration.

The radiological personnel also argued that it could be difficult for the GPs to make decisions regarding diagnostic imaging. This was in part because the clinical tests used today have limitations that make providing a definite diagnosis difficult. Clinical assessment of a patient, in combination with tight time restraints, can be difficult.

Finally, health care workers and the management’s lack of focus on, and *attitudes towards guidelines* was perceived to affect guideline adherence by both groups interviewed, as this statement from one of the radiological personnel shows:

*“But it hasn’t been any focus on [the guideline] from the higher ups anyway. We may not be that good at referring to these kinds of guidelines in general.”*

The results indicate that there may be a general opposition towards guidelines among some GPs, due to guideline overload. There also seems to be a lack of focus on guidelines among radiological departments’ management. Many of these guidelines have changed rapidly, and the GPs argued that there could be too much information to be able to adhere to all the different guidelines.

## Discussion

Our findings demonstrate that the informational meetings were the most successful part of the implementation, because they created an awareness of the guidelines’ existence and their potential to make good decisions with respect to examinations, and to confirm that GPs and radiologists’ practice was mostly correct. This is in accordance with the literature, in which informational meetings have been seen to have great potential for change in guideline adherence [29–31]. Studies have reported increases in guideline adherence between 9 and 82% when applying informational meetings in different forms [11, 12, 29, 31, 32]. The participants were also mostly happy with the contents of the informational meetings, which may also have contributed to the experience that this was the most successful part of the implementation. According to IF, the content quality is also an important moderator for the outcome of an implementation [24], and in our study the quality of the content may have had a positive effect of the perceived outcome .

The two passive parts of the implementation (physical copy of short version and access to digital resources) did not seem to change guideline use and were experienced as the least successful part of the implementation. GPs only used the guideline for a short while before it was lost, and the radiological personnel did not use it at all, which thereby did not spur any major change.

This corresponds partly with the literature, in which passive forms of implementation have shown some potential for change in guideline use and adherence. In our study no lasting change was found, while other studies have demonstrated that what yielded the least change was simply publishing the guideline (no significant change) [9]. The passive method that showed greater change came from a combination of postal dissemination and dissemination of a local study showing the benefits of the guideline (30% reduction in referrals contradicting guidelines) [33].

The experience that only parts of the implementation were somewhat successful can also help explain the experience from both GPs and radiological personnel with lack of major changes in guideline use, reference pattern, or communication between primary and secondary care. Rather than using the guideline as a decision support tool, GPs used the guideline in discussions with patients who wanted diagnostic imaging, since this is when they reckon the guideline was most useful. Radiological personnel reported not using the guideline after the implementation. This was partly due to many of the radiological personnel not participating in the informational meetings, partly to poor placement of the physical short version. Some difficulties finding the online resources influenced the lack of guideline use.

The lack of guideline use might also be related to how the implementation was delivered, which according to IF is one of the affecting factors for an implementations outcome [24]. It could be problematic that the participants had not seen a need for the information and sought it out themselves as a response to a need. The informational meetings were offered to a representative for the participating municipalities, and it may be that not all participants were interested in this guideline. Several of the participants in the focus group interviews also mentioned that they considered the informational meetings as a confirmation of their practice being mostly correct, which may indicate a perceived lack of need to change their practice for those participants.

According to IF, another factor that can influence the perceived outcome of an implementation is the exposure, or dose, of the implementation [24]. This means how much of the target group is covered, how often, and for how long. During the implementation process it was difficult to cover the entire target group. It was especially challenging with the radiological personnel, because meetings with the entire department are challenging to arrange. Therefore, in the evaluation many of the radiological personnel in the focus group interviews had not attended the informational meetings. This may have influenced the process and perceived outcome of the implementation. It was also mentioned during the focus group interviews that the information should have been conveyed at least one more time, a while after the original meeting. This indicates that the exposure, or dose, was not optimal for the target group, which also can have influenced the outcome.

This is in combination with factors CFIR refers to as the inner setting (e.g, networks and communications, structural characteristics, culture and implementation climate) and the outer setting (e.g, patient needs and resources, peer pressure, and external policies and incentives) which together make changes in practice difficult [27]. For example, it was mentioned that communication between radiologists and referrers, other than by phone was difficult and time consuming. This has also been showed in earlier studies, where it has been reported as an important factor when examinations have been performed in which radiologists doubt the usefulness of the examination [34]; radiographers have also reported similar experiences about contacting the referring physician being time consuming [35].

Our study’s participants suggested that the electronic referral system could have an option for direct communications between referrers and radiological personnel; they also mentioned that common meetings between the two groups could improve communication, when for example, they could discuss guidelines and local protocols in the radiology departments (hospital and privately run institutions).

Like the CFIR, we found the patient demand factor as a key factor in the implementation process and its perceived outcome [27]. Participants experienced that patient demand influenced guideline use, because patients could leave bad reviews online if they were dissatisfied with the consultation, and would change to another GP if they did not receive a referral for diagnostic imaging. Patient demands could be initiated by stories in the media depicting patients being saved because they received diagnostic imaging in time. This, in combination with a lack of time for both referrers and radiological personnel made guideline use difficult. This problem is mentioned in other studies, in which lack of time was one of the largest barriers to guideline use and proved difficult to overcome [25, 36–40].

Two other factors mentioned during the focus group interviews related to the construct of the inner setting in CFIR [27]. First, both GPs and radiological personnel view professional autonomy as one of the most important parts of their job. This has also been identified previously, that health professionals have been worried about being stuck performing cook-book medicine and clinical judgement was being lost [41]. Second, the lack of a focus on guidelines from radiological departments’ management (both in public hospitals and private institutions) and an excessive focus on guidelines by the government for the GPs. The lack of focus on guidelines from management lead to a lack of awareness and use of guidelines for the radiological personnel. For the GPs the excessive focus on, and amount of, guidelines led to them not being able to relate to all of them.

All these factors combined can help explain the lack of changes experienced in practice by both the GPs and the radiological personnel. Our study indicates that in the Norwegian context the implementation was unable to reduce the existing barriers to guideline use and adherence. The context of primary care and radiology may also have influenced the findings, since providing educational material, and educational meetings has been found more effective in other contexts [29–31]. Had the implementation been changed to either be a digital reminder message, or just have a repeat of the meetings this may also have shown a different outcome. Our experiences can, however, be of great value for improved implementation of guidelines and for other approaches that can spur behavioral changes for appropriate care. On the other hand, the success of the implementation cannot be stated as of yet, since these results are based on the participants perceptions, and could be biased.

This study will be followed up by a quantitative study that is an evaluation of the referral rate for the diagnostic imaging covered by the implementation. This will show another aspect of the outcome of the implementation, in the shape of any significant changes in the use of diagnostic imaging.

### Limitations

One limitation of this study was that the guideline was already introduced, and this implementation was technically a re-implementation. However, the official introduction consisted of publishing the guideline on a web page and a postal dissemination. Hence, it was not an implementation interacting with the target groups.

The fact that several of the participating GPs and radiological personnel had not attended the earlier informational meetings limits the evaluation of these meetings. This was especially the case for the radiological personnel, because most of their participants in the focus group interview had not participated in the informational meetings. However, since this only applied to one part of the implementation content, this may not have had a major impact on the overall evaluation of the implementation.

Finally, the theoretical frameworks used to develop the questions and as background for the evaluation have their own limitations. CFIR was originally developed for research on more specific and focused interventions, rather than on more complex interventions situated in different contexts, like this study [42]. Adapting CFIR to this kind of implementation can, therefore, be challenging [42]. However, in this study it was not used directly but rather as a guide and basis for the planning stages and the implementations evaluation. The limitations when using self-reported data to measure IF are related to challenges with accuracy and validity [43]. The data can be distorted because the participants’ reports may be biased by their feelings towards the implementers, a tendency to over-report adherence, and a desire to provide a positive assessment of their adherence to guidelines [43]. This could also be the case in this study, even if the participant seemed to give an honest opinion regarding the implementation and assessment of their adherence.

## Conclusions

In conclusion, we found that the participants only deemed the informational meetings and digital resources as successful, and there were small or no changes in practice. Several factors perceived to influence the implementation process and outcome by both target groups were also identified (lack of systems for direct communication, and pressure from patients). Some of the factors identified were only mentioned by one of the target groups, such as guideline overload (GPs), and lack of focus on guidelines, lack of regulatory body for x-ray referrals (radiological personnel).

The results also show that even tailored implementation strategies have difficulties reaching the entire target group and spur a change in practice when existing barriers to guideline use are not overcome, resulting in lack of change experienced within the target groups.

Although these results stem from a specific project in Norway, they reveal important factors and mechanisms that can be transferred to others and can be of great value for policy makers and healthcare organizations involved in implementing guidelines and for other approaches reinforcing appropriate care.

Based on our results we recommend that further work in guideline implementation continue to utilize an active approach that engages the target group more in the intervention design and implementation. Moreover, further steps should be taken to overcome the identified barriers to guideline use and adherence. The actual need for change, and thereby implementation, should also be assessed before starting an implementation.

## Supplementary information

**Additional file 1.** Interview guide.

## Data Availability

The datasets used and/or analyzed during the current study are available from the corresponding author on reasonable request.

## Abbreviations

CFIR: Consolidated Framework for Implementation Research
CPOE: Computerized Physician Order Entry
CR: Conventional Radiography
GP: General Practitioner
IF: Implementation Fidelity
NAV: Norwegian Labour and Welfare Services
NEL: Norwegian Clinical Manual
NSD: Norwegian Social Science Data Services
