# Supplementary material for: Health care personnel’s perception of guideline implementation for musculoskeletal imaging: a process evaluation
Source: BMC Health Serv Res. 2020 May 11;20:397. doi: 10.1186/s12913-020-05272-9 (PMC7212587; doi:10.1186/s12913-020-05272-9)
Supplement: Supplementary file 1 — Additional file 1. Interview guide. [file 12913_2020_5272_MOESM1_ESM.docx]

Interview guide

Subject 1- The intervention

- What has it been like using the guideline in general?
- How has the use of the guideline changed?
- What has it been like to incorporate the guideline recommendations to your day-to-day work?
- How would you rate the quality of the material provided (physical short version, educational meetings)?
- How do you feel that the education and written material affected your knowledge on the field?
- How would you deem the accessibility of the guideline and/or further information regarding the guideline?

Subject 2 – Outer setting

- Have you used the guideline in meetings with challenging patients? If yes, how? If no, why not?
- Do you experience any changes in referrals/demands of documentation from NAV? If yes, what changes?

Subject 3 – Inner setting

- Have you experienced any changes in communication between radiological personnel and referrers after the guideline implementaiton (have you given/received more feedback? Has any referrals been denied based on guideline recommendations? Why/why not?)?
- Have you experienced changes in the quality of the referrals? (changes in information given, higher certainty of modality, confirm disease vs elimination of conditions, etc)
- Have you experienced any changes in the number of referrals? If yes, which? (more referrals, less referrals?
- Do you feel you have changed you referral pattern? If so, what changes have been made? (Refer to other modalities than before, changes in what patients are referred?)? If no, why not?
- If anything could have been done differently to strengthen the knowledge of the guideline, what changes would you recommend?
- Is there anything we have not asked that you feel it is important that we know?
